# The critical care management of poor-grade subarachnoid haemorrhage

**DOI:** 10.1186/s13054-016-1193-9

**Published:** 2016-01-23

**Authors:** Airton Leonardo de Oliveira Manoel, Alberto Goffi, Tom R. Marotta, Tom A. Schweizer, Simon Abrahamson, R. Loch Macdonald

**Affiliations:** 1 St. Michael’s Hospital, 30 Bond Street, Toronto, ON M5B 1 W8 Canada; 2 Keenan Research Centre for Biomedical Science of St. Michael’s Hospital, 30 Bond Street, Toronto, ON M5B 1 W8 Canada; 3 Toronto Western Hospital MSNICU, 2nd Floor McLaughlin Room 411-H, 399 Bathurst Street, Toronto, ON M5T 2S8 Canada

## Abstract

Aneurysmal subarachnoid haemorrhage is a neurological syndrome with complex systemic complications. The rupture of an intracranial aneurysm leads to the acute extravasation of arterial blood under high pressure into the subarachnoid space and often into the brain parenchyma and ventricles. The haemorrhage triggers a cascade of complex events, which ultimately can result in early brain injury, delayed cerebral ischaemia, and systemic complications. Although patients with poor-grade subarachnoid haemorrhage (World Federation of Neurosurgical Societies 4 and 5) are at higher risk of early brain injury, delayed cerebral ischaemia, and systemic complications, the early and aggressive treatment of this patient population has decreased overall mortality from more than 50 % to 35 % in the last four decades. These management strategies include (1) transfer to a high-volume centre, (2) neurological and systemic support in a dedicated neurological intensive care unit, (3) early aneurysm repair, (4) use of multimodal neuromonitoring, (5) control of intracranial pressure and the optimisation of cerebral oxygen delivery, (6) prevention and treatment of medical complications, and (7) prevention, monitoring, and aggressive treatment of delayed cerebral ischaemia. The aim of this article is to provide a summary of critical care management strategies applied to the subarachnoid haemorrhage population, especially for patients in poor neurological condition, on the basis of the modern concepts of early brain injury and delayed cerebral ischaemia.

## Background

Aneurysmal subarachnoid haemorrhage (SAH) is a complex neurovascular syndrome with profound systemic effects and is associated with high disability and mortality [1]. Despite a 17 % decrease in case fatality in the last three decades associated with improved management strategies, 30-day mortality and before-admission death rate unfortunately are still high, around 35 % and 15 %, respectively [2].

Outcomes after SAH can vary significantly, from full recovery to severe disability or death, depending on the severity of the initial bleed and potential complications typically happening in the first 2 weeks after the haemorrhage [3]. The level of consciousness is considered the most important early predictor of outcome [4–6]. Patients with a normal level of consciousness have a low risk of mortality. Patients admitted with a depressed level of consciousness have higher risk of death and disability, although improved outcomes have also been shown in this group of patients in the last decades. For these reasons, patients presenting with a Glasgow Coma Scale (GCS) score of less than 13 have traditionally been defined as having poor-grade SAH (classified as grade 4 and 5 according to the Hunt and Hess [4] or the World Federation of Neurosurgical Societies (WFNS) grading scales [5] or more recently as VASOGRADE-Red [6]).

Poor outcomes are usually secondary to early brain injury (EBI) or to delayed cerebral ischaemia (DCI). EBI refers to the acute consequences of SAH-associated sudden increase of intracranial pressure (ICP), which can cause decreased cerebral perfusion and transient global cerebral ischaemia. The global cerebral ischaemia can result in transient loss of consciousness or progressive intracranial hypertension. Subarachnoid blood itself can also damage the brain. DCI is a multifactorial entity often responsible for poor outcome after SAH in patients who survive the initial haemorrhage. Clinically, it is characterised by a change in neurological function that manifests most often between days 3 and 14 after haemorrhage. Recently, DCI was defined as a change in level of consciousness (decrease of 2 points in the GCS or an increase in 2 points in the National Institute of Health Stroke Scale) or development of new focal deficit lasting for at least 1 hour and not explained by other factors (e.g., systemic complications and hydrocephalus) [7]. DCI is believed to be due to a combination of factors such as angiographic vasospasm, cortical spreading ischaemia, microthrombosis, and microcirculation vasoconstriction. In this review, we will discuss the management of patients with poor-grade SAH on the basis of the current concepts of EBI and delayed cerebral ischaemia.

## Search strategy

A PubMed search for articles published until May 2015 was performed by using the terms “Subarachnoid Hemorrhage” [Mesh] AND (“poor-grade” [Title/Abstract] OR “high-grade” [Title/Abstract]), which returned 236 articles. Additionally, the reference lists of the most recent guidelines on the management of SAH were searched [8–10]. Lastly, the authors’ personal databases were used as an additional source for this review.

## 1. Initial management: medical stabilisation, prevention of re-bleeding, and control of intracranial pressure

**Fig. 1:**
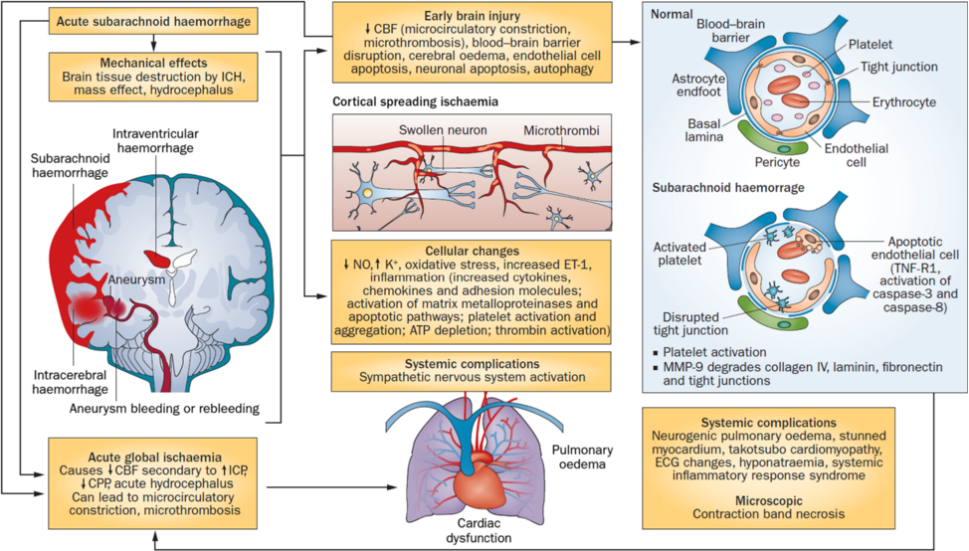
Early pathophysiology of subarachnoid haemorrhage. Acute haemorrhage from an aneurysm can physically damage the brain and lead to acute transient global ischaemia. Transient global ischaemia secondary to increased intracranial pressure can also trigger sympathetic nervous system activation, leading to systemic complications. The contribution of each process to the pathophysiology is unknown, but transient global ischaemia and subarachnoid blood result in early brain injury, characterised by microcirculation constriction, microthrombosis, disruption of the blood–brain barrier, cytotoxic and vasogenic cerebral oedema, and neuronal and endothelial cell death. *CBF* cerebral blood flow, *CPP* cerebral perfusion pressure, *ECG* electrocardiographic, *ET-1* endothelin-1, *ICH* intracranial haemorrhage, *ICP* intracranial pressure, *MMP-9* matrix metalloproteinase-9, *NO* nitric oxide, *TNF-R1* tumour necrosis factor receptor 1. First published in *Nature Reviews Neurology* [98]

During aneurysmal SAH, extravasation of high-pressure arterial blood in the subarachnoid space (and often into the brain parenchyma and ventricles) is associated with a sudden ICP increase that, if severe and sustained, may compromise cerebral perfusion, causing global cerebral ischaemia and EBI (Fig. 1). If the haemorrhage does not stop, acute cardiopulmonary instability associated with intracranial hypertension or compromised cerebral blood flow (CBF) leads to patient death before hospital admission. In patients who survive the initial haemorrhage, re-bleeding is the most severe early complication; the reported incidence is up to 15 % in the first 24 hours, and the fatality rate is approximately 70 % [11–13]. Patients with poor-grade SAH are at higher risk of re-bleeding [14]. Initial management therefore should focus on strategies aimed to prevent re-bleeding and to control ICP.

Early aneurysm repair is generally considered the most important strategy to reduce the risk of aneurysm re-rupture [15]. However, evidence for optimum timing of treatment is limited, and it is unclear whether ultra-early treatment (less than 24 hours) is superior to early aneurysm repair (within 72 hours). A recently published retrospective data analysis comparing ultra-early treatment with repair performed within 24–72 hours after haemorrhage suggests that aneurysm occlusion can be performed safely within 72 hours after aneurysm rupture [16]. The American Heart Association/American Stroke Association [9] recommend as a Class IB Recommendation that “surgical clipping or endovascular coiling of the ruptured aneurysm should be performed as early as feasible in the majority of patients to reduce the rate of re-bleeding after SAH”. This recommendation for timing of aneurysm intervention is corroborated by the European Stroke Organization Guidelines for the Management of Intracranial Aneurysms and Subarachnoid Haemorrhage [10], which stated that “aneurysm should be treated as early as logistically and technically possible to reduce the risk of re-bleeding; if possible it should be aimed to intervene at least within 72 hours after onset of first symptoms”.

The results from an ongoing trial only enrolling patients with poor-grade SAH may help answer the question of whether early treatment (within 3 days) is associated with improved outcome compared with intermediate (days 4–7) or late (after day 7) treatment [17].

The choice of treatment modality between surgical clipping and endovascular coiling is a complex endeavour, which requires the expertise of an interdisciplinary team, including neurointensivists, interventional neuroradiologists and neurovascular surgeons. For aneurysms considered to be equally treatable by both modalities, the endovascular approach is superior, being associated with better long-term outcomes [18–20]. Randomised trials of clipping versus coiling included mostly good-grade patients, leading to controversy as to whether their results apply also to poor-grade patients. Retrospective data on clipping and coiling in poor-grade patients seem to suggest that surgical clipping and endovascular are equally effective [21]. An early and short course of an anti-fibrinolytic drug such as tranexamic acid, initiated as soon as the radiological diagnosis of SAH is established and stopped within 24–72 hours, has been associated with decreased rate of ultra-early re-bleeding and a non-significant improvement in long-term functional outcome [22]. This approach remains controversial [23], and short-term administration of tranexamic acid to prevent re-bleeding is being further studied in a multicentre randomised trial (Dutch Trial Registry number NTR3272) [24]. Another medical intervention applied to prevent aneurysm re-rupture is the avoidance of extremes of blood pressure. The American Heart Association/American Stroke Association [9] and the Neurocritical Care [8] guidelines suggest keeping the mean arterial blood pressure below 110 mm Hg or systolic blood pressure below 160 mm Hg (or both) in the presence of ruptured unsecured aneurysm. The European guidelines are less aggressive and suggest keeping the systolic blood pressure below 180 mm Hg [10]. These parameters should not be used after aneurysm treatment, when spontaneously high blood pressure may be beneficial [25].

Intracranial hypertension (ICP of at least 20 mm Hg) is a relatively common complication of SAH, especially in patients presenting with poor neurological condition [26–28]. Multiple factors such as cerebral oedema, intraparenchymal haematoma, acute communicating hydrocephalus, intraventricular haemorrhage, aneurysm re-rupture, complications related to aneurysm treatment, EBI, and DCI can contribute to the development of intracranial hypertension [29]. High ICP is associated with severe derangements of cerebral metabolism [30], increased risk of neurological deterioration [25], and poor outcome, especially if refractory to medical treatment [29, 31]. ICP of greater than 20 mm Hg is an independent predictor of severe disability and death in aneurysmal SAH [30].

Principles of management of intracranial hypertension after SAH have been traditionally adopted from traumatic brain injury (TBI) literature [32] and are not specifically designed for the SAH population. However, these two entities are different from a pathophysiological perspective, and the use of therapies tested in patients with TBI in the SAH population is controversial. Currently, the role of therapies such as hyperosmolar agents, hypothermia, barbiturates, and decompressive craniectomy is not well established in SAH patients with intracranial hypertension refractory to first-line treatments.

The initial approach to raised ICP includes head of bed elevation (between 30° and 45°) to optimise cerebral venous drainage, normoventilation (arterial partial pressure of carbon dioxide (PaCO_2_): 35–40 mm Hg) [33], use of sedation and analgesia to achieve a calm and quiet state (Richmond Agitation Sedation Scale score of −5 or Sedation-Agitation Scale score of 1), and surgical intervention in the presence of mass-occupying lesions [34]. The use of neuromuscular blocking agents is sometimes applied to prevent ICP surges during tracheal suctioning and physiotherapy; however, the role of these drugs for ICP management is not well established, and some authors suggest that they may be more deleterious than beneficial [35]. If ICP remains elevated despite these interventions, a short course (less than 2 hours) of hyperventilation (PaCO_2_ of 30–35 mm Hg) might be considered while new brain imaging is obtained and other interventions are planned and initiated [36–38].

Cerebrospinal fluid (CSF) drainage is a mainstay in ICP management of patients with SAH, especially when hydrocephalus is present [39]. Acute hydrocephalus is common in SAH, and approximately 50 % of patients are affected on admission [40]. When hydrocephalus is associated with a decreased level of consciousness, an external ventricular drain (EVD) should be inserted to allow CSF drainage and ICP monitoring. EVD insertion before aneurysm treatment has been shown to be safe and not associated with increased risk of aneurysm re-rupture [40, 41], if accompanied by early aneurysm repair. Additionally, when EVD insertion is performed before aneurysm repair, CSF drainage should be practiced with caution because rapid and aggressive CFS drainage can increase transmural pressure, increasing the risk of aneurysm re-rupture [41, 42]. Interestingly, approximately 30 % of patients with poor-grade SAH improve neurologically after EVD insertion and CSF drainage. These responders have a functional outcome similar to that of good-grade (WFNS I–III) patients [39].

Hyperosmolar agents, such as mannitol and hypertonic saline, are usually considered when the above strategies fail to control ICP, although their role on clinical outcome in the SAH population is not well established. We could not identify any study addressing the role of mannitol in the management of raised ICP in the SAH population; for hypertonic saline, we found only case series [43–46] and a small placebo-controlled trial in patients with raised but stable ICP [47]. In these studies, hypertonic saline was effective to control ICP and improved CBF [43–47] and may improve outcome in the poor-grade population [43].

The last line of treatment includes the use of barbiturates, induced hypothermia, and decompressive craniectomy [38, 48]. Therapeutic hypothermia has been shown to be effective to control ICP in SAH but has not been associated with improved functional outcome and reduced mortality rates in patients with poor-grade SAH [49]. The association of barbiturate coma and mild hypothermia (33–34 °C, median treatment of 7 days) was studied in 100 SAH (64 poor-grade) patients with intracranial hypertension refractory to other medical interventions [50]. Approximately 70 % of patients were severely disabled or dead at 1 year, and more than 90 % of patients developed medical complications associated with the hypothermia/barbiturate treatment (i.e., electrolyte disorders, ventilator associated pneumonia, thrombocytopenia, and septic shock). Decompressive craniectomy is another possible strategy for refractory ICP management in patients with SAH. Poor-grade patients are more commonly exposed to this rescue therapy than patients with good-grade SAH [51, 52]. Decompressive craniectomy has been associated with decreased mortality [53], significant reduction of ICP [34], improved cerebral oxygenation [54, 55], and improved cerebral metabolism [56]. However, most patients undergoing decompressive craniectomy due to refractory ICP have poor outcome, with severe disability or death [56]. Many authors suggest that, if any benefit can be achieved with decompressive craniectomy, this may be best obtained when the procedure is performed early (within 48 hours from the bleeding) [52] and in the absence of radiological signs of cerebral infarction [51]. Finally, in poor-grade patients with large intraparenchymal or Sylvian fissure haematomas usually from middle cerebral artery aneurysms, prophylactic decompressive craniectomy should be considered [34].

It is important to mention that long-term outcome after acute brain injury is markedly improved when patients are managed in a dedicated neurologic/neurosurgical intensive care unit (ICU) [57, 58]. Especially after SAH, outcome is affected by hospital caseload, and better outcomes happen in high-volume centres (centres treating more than 60 patients per year) [59]. Six-month mortality is inversely associated with hospital annual caseload; there is a 24 % reduction in mortality for each 100 patients admitted per year [60]. Regardless of initial grade, early transfer to a high-volume centre is safe and cost-effective and should be pursued [61–63].

## 2. Prevention, detection, and treatment of delayed cerebral ischaemia

**Fig. 2:**
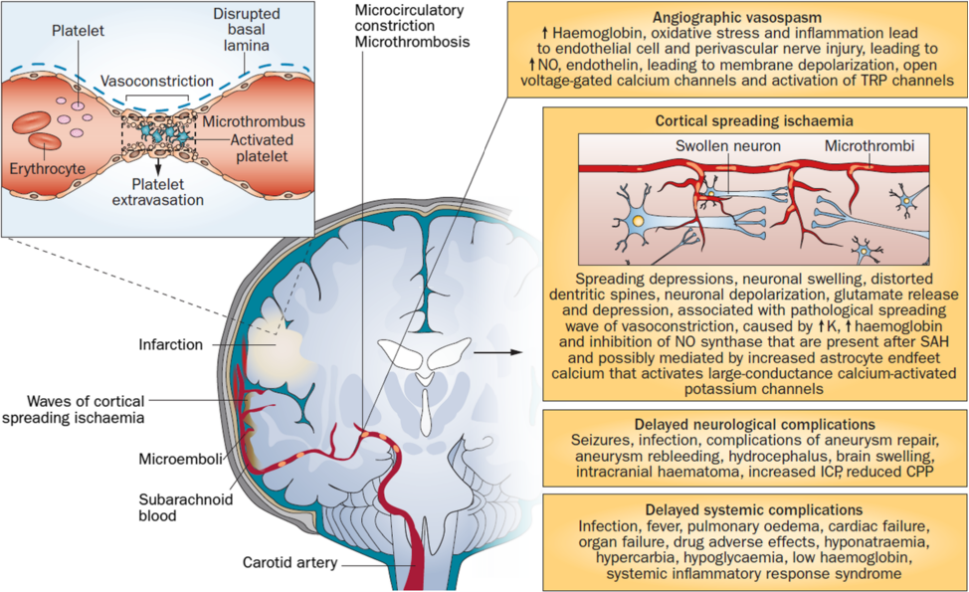
Pathophysiological processes in delayed cortical ischaemia. Key processes include angiographic vasospasm, microcirculatory constriction and formation of microthrombi, and waves of cortical spreading ischaemia, all of which can contribute to cerebral infarction. Delayed effects of the early brain injury such as neuronal and endothelial cell apoptosis, and systemic complications, can also occur. *CPP* cerebral perfusion pressure, *ICP* intracranial pressure, *NO* nitric oxide, *SAH* subarachnoid haemorrhage, *TRP* transient receptor potential. First published in *Nature Reviews Neurology* [98]

Delayed neurological deterioration occurs frequently in the first 2 weeks after SAH. Common causes of this deterioration include neurological events such as progression of EBI, hydrocephalus, seizures, ischaemia, and systemic conditions, such as fever and infections, respiratory failure, and electrolyte abnormalities. Any delayed neurological deterioration presumed to be related to ischaemia that persists for more than 1 hour and cannot be explained differently has been defined as DCI [7] (Table 1). DCI occurs in up to 30 % of SAH patients surviving the initial haemorrhage. It can present as an acute or insidious change in the level of consciousness or as a focal neurological symptom, such as aphasia or hemiparesis, or as both. These symptoms can be reversible if treated promptly and aggressively; otherwise, DCI tends to progress to cerebral infarction, which is associated with higher rates of disability and mortality. Traditionally, DCI has been considered to be related to a cerebral vasoconstriction (angiographic vasospasm) that begins approximately 3 days and peaks 1 week after the haemorrhage and starts resolving after 2 weeks [64]. However, recent evidence suggests that DCI is a complex, multifactorial syndrome, which can include additional pathophysiologic processes beyond angiographic or sonographic vasospasm (Fig. 2 and Table 2) [65]. DCI may also occur in cerebral territories without evidence of angiographic vasospasm [66]. EBI (defined as brain injury developing in the first 72 hours after haemorrhage) has significant impact on likelihood and severity of subsequent ischaemic changes [67, 68]. For example, poor-grade patients, who have worse EBI, as well as patients who lose consciousness at the time of SAH (and therefore have at least a short episode of transient global cerebral ischaemia) have increased risk of DCI [67, 68].

**Table 1:** The current definitions of early brain injury, delayed cerebral ischaemia, and cerebral infarction

A. Early brain injury is the acute consequence of subarachnoid haemorrhage (SAH) that leads to transient global cerebral ischaemia following the aneurysm rupture. During aneurysmal rupture, arterial blood leaks under high pressure into the subarachnoid space and often into the brain parenchyma and ventricles. There is an acute and sharp increase in the intracranial pressure (ICP) that may rise high enough to compromise cerebral perfusion, causing global cerebral ischaemia. This acute drop in cerebral perfusion pressure usually produces loss of consciousness. The initial cerebral injury (i.e., early brain injury) is the combined result of transient global cerebral ischaemia and the effects of the subarachnoid blood itself. B. Global cerebral ischaemia: As discussed above, the aneurysm rupture leading to SAH can increase the ICP to cause global cerebral ischaemia. If the haemorrhage does not stop, the patient dies before hospital admission and this is usually due to acute cardiopulmonary changes associated with the high ICP or due to brain death related to the compromised cerebral blood flow. Re-bleeding remains the most important complication in the hours following the initial bleed. Therefore, the initial management should focus on strategies to prevent aneurysm re-bleeding and to control ICP. C. Delayed cerebral ischaemia (DCI) is defined as “the occurrence of focal neurological impairment (such as hemiparesis, aphasia, apraxia, hemianopia, or neglect), or a decrease of at least 2 points on the Glasgow Coma Scale (either on the total score or on one of its individual components, such as eye, motor on either side, or verbal). This should last for at least 1 hour, is not apparent immediately after aneurysm occlusion, and cannot be attributed to other causes by means of clinical assessment, CT or MRI scanning of the brain, and appropriate laboratory studies” [7]. DCI remains the most significant cause of long-term disability and mortality in patients who survive the initial haemorrhage to reach definitive aneurysm treatment [163]. In those patients who survive the initial bleed to reach medical assistance, the degree of brain injury associated with transient global cerebral ischaemia is variable. However, the main factor associated with the degree of injury and long-term outcome is ultimately the level of consciousness. Patients with small haemorrhages at the time of aneurysm rupture usually do not develop transient cerebral ischaemia and do not lose consciousness; however, they are still at risk of DCI [164]. On the other hand, patients who transiently lose consciousness have probably had a transient global ischaemic event and are at a higher risk of DCI [67]. D. Cerebral infarction caused by DCI is defined as “the presence of cerebral infarction on computed tomography or magnetic resonance scan of the brain within 6 weeks after SAH, or on the latest scan made before death within 6 weeks, or proven at autopsy, not present on the computed tomography or magnetic resonance scans between 24 and 48 hours after early aneurysm occlusion, and not attributable to other causes such as surgical clipping or endovascular treatment. Hypodensities on computed tomography imaging resulting from ventricular catheter or intraparenchymal haematoma should not be regarded as cerebral infarctions from DCI” [7].	

*CT* computed tomography, *MRI* magnetic resonance imaging

**Table 2:** Facts that challenged the concept of angiographic vasospasm as the main factor leading to delayed cerebral ischaemia

A. Approximately 70 % of patients with subarachnoid haemorrhage (SAH) will develop some degree of angiographic vasospasm within 2 weeks of haemorrhage [64, 165]; however, only 30 % will develop symptoms (i.e., delayed cerebral ischaemia, or DCI) [88]. B. DCI-associated cerebral infarct is an independent factor for poor outcome after SAH [166]; however, cerebral infarction can happen asymptomatically [88] or in vascular territories not affected by vasospasm [167]. C. Large-vessel angiographic vasospasm detected by modalities such as transcranial Doppler has a poor temporal relationship with the development of DCI [167]. D. There is no evidence that nimodipine decreases the rate of angiographic vasospasm or promotes cerebral vasodilation; however, it remains the sole pharmacological intervention proven to improve outcomes from DCI [108, 111]. E. There is an important dissociation between vasospasm-related morbidity and functional outcome after SAH [168, 169]. F. The prevention and treatment of angiographic vasospasm do not necessarily translate into improved outcome [169].	

Cortical spreading ischaemia (CSI) is a wave of depolarisation in the grey matter that propagates across the brain at 2–5 mm/minute [69, 70], leading to depression in evoked potentials and spontaneous electroencephalogram activity. The use of invasive subdural electrocorticographic monitoring combined with regional CBF measurements has shown that CSI can occur isolated or in clusters, and the depolarisation waves are associated with profound cortex hypoperfusion secondary to vasoconstriction [71]. The vast majority of cortical spreading depolarisation waves usually happen in the first 2 weeks after aneurysm rupture, and 75 % of all CSIs recorded occur between the fifth and seventh day post-bleeding [72]. In a prospective multicentre study, Dreier et al. [73] assessed the incidence and timing of spreading depolarisations and DCI after SAH. Eighteen SAH patients requiring craniotomy for aneurysm treatment were monitored for up to 10 days with subdural electrodes. Cortical spreading depolarisations were detected in 13 patients (72 %). DCI was detected in seven patients and was time-locked to a sequence of recurrent spreading depolarisations in all seven cases. Additionally, delayed ischaemic strokes verified by serial computed tomography (CT) scans or magnetic resonance imaging occurred in the recording area in four patients. In another prospective study, using a novel subdural opto-electrode technology for simultaneous laser-Doppler flowmetry and direct current-electrocorticography, combined with measurements of tissue partial pressure of oxygen, Dreier et al. [71] studied 13 patients with SAH. Isolated spreading depolarisations were detected in 12 of those. These waves of depolarisations were associated with physiological, absent, or inverse regional CBF responses. Normal haemodynamic response was associated with tissue hyperoxia, whereas inverse response led to tissue hypoxia. Five patients presented clusters of prolonged spreading depolarisations with persistent depressions. These clusters of spreading depolarisations were closely associated with structural brain damage as observed by neuroimaging. Similarly, Bosche et al. [72] have reported low cerebral measurements of tissue partial pressure of oxygen occurring during clusters of spreading depolarisations.

Microthrombosis is common after SAH [74]. Subarachnoid blood and blood products activate inflammatory pathways, along with tissue factor in the microcirculation of cerebral vessel wall, leading to endothelial cell activation and damage, which in turn cause mural thrombus formation and release of microemboli [75]. Markers of increased activity of the coagulation cascade have been associated with DCI, cerebral infarction, and poor outcome [76]. For example, in a group of 90 patients with SAH, early (within 3 days of SAH onset) elevated concentrations of von Willebrand factor were associated with poor outcome (crude odds ratio (OR) = 4.6, 95 % confidence interval (CI) 2.0–10.9; adjusted OR = 3.3, 95 % CI 1.1–9.8), ischaemic events (crude hazard ratio (HR) = 2.3, 95 % CI 1.1–4.9; adjusted HR = 1.8, 95 % CI 0.8–3.9), and occurrence of spontaneous DCI (crude HR = 3.5, 95 % CI 0.9–13.1; adjusted HR = 2.2, 95 % CI 0.5–9.8). The hypothesis is that this early elevation in von Willebrand factor levels probably reflects the formation of microthrombi in the cerebral circulation [77, 78]. Autopsy studies have shown that patients who developed DCI-related cerebral infarction had significantly more microthrombi compared with SAH patients who died because of re-bleeding or acute hydrocephalus [75, 79].

Haptoglobin is a complex tetramer glycoprotein, consisting of two α and two β chains, synthesised mainly by the liver [80]. It is an acute-phase protein that increases in plasma during major stress situations, such as sepsis, burns, and major trauma. Some recent studies have suggested that the haptoglobin α1-α1 isoform could be protective after SAH [81–83]. Haptoglobin binds free extracellular haemoglobin, which reduces free haemoglobin ability to generate oxygen-free radicals and therefore interferes in one of the possible pathophysiological pathways of angiographic vasospasm (i.e., haemoglobin-mediated oxidative stress) [82].

Kantor et al. [82] found, in a cohort of 193 patients with SAH, that the haptoglobin α2-α2 isoform was associated with worse functional outcome at 3 months when compared with the α1-α1 genotype. The haptoglobin α2-α2 isoform has a lower affinity for binding haemoglobin and possibly inhibits haptoglobin-haemoglobin clearance because of its larger size [84]. The α2-α2 genotype remained significantly associated with worse functional outcome (OR 4.138; *P* = 0.0463) after adjustment for age, sex, Fisher grade, and Hunt and Hess grade. A previous study had already shown that haptoglobin α2-α2 genotype was associated with higher rates of angiographic vasospasm by transcranial Doppler (TCD) and conventional angiography performed between days 3 and 14 after SAH [81]. A recent study by Leclerc et al. [83] showed, in a cohort of 74 patients with SAH, that haptoglobin α2-α2 genotype was an independent risk factor for the development of focal and global angiographic vasospasm and also predictive of unfavourable functional outcomes and mortality.

The hypothesis is that patients with haptoglobin α2-α2 genotype do worse because of reduced CSF clearance of haemoglobin, increased reactive oxygen species, and therefore development of more inflammation. This hypothesis is corroborated by an experimental model of SAH, which showed that mice expressing human α2-α2 haptogobin developed more severe angiographic vasospasm and increased macrophage/neutrophil counts in the CSF after SAH, when compared with wild-type α1-α1 haptogobin-expressing mice [85]. Although there is no clinical intervention directly designed to address this important recent finding on the pathophysiology of SAH, the genetic effect on outcome after SAH may increase our knowledge of the disease.

### Delayed cerebral ischaemia monitoring. Triggers for detection and confirmation of delayed cerebral ischaemia in sedated or poor-grade patients

**Fig. 3:**
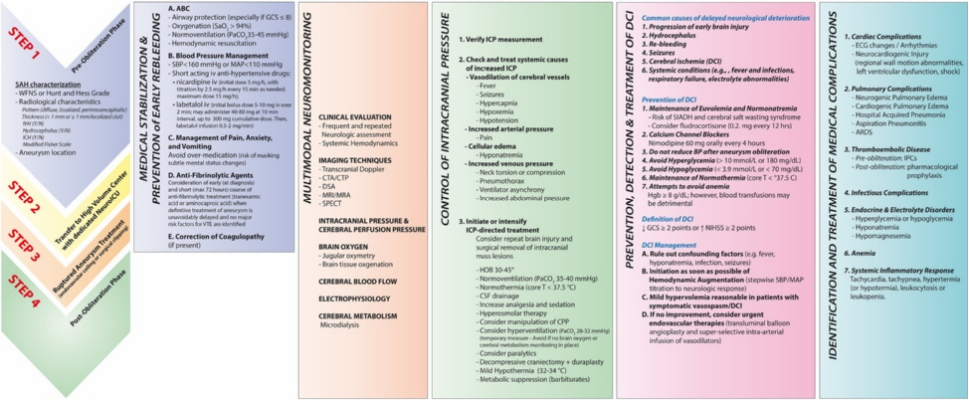
Summary of a possible approach for the management of subarachnoid haemorrhage patients in poor neurological condition. *ARDS* acute respiratory distress syndrome, *BP* blood pressure, *CPP* cerebral perfusion pressure, *CSF* cerebrospinal fluid, *CTA/CTP* computed tomography angiography/computed tomography perfusion, *DCI* delayed cerebral ischaemia, *DSA* doxyl stearic acid, *ECG* electrocardiogram, *GCS* Glasgow Coma Scale, *Hgb* haemoglobin, *HOB* head of bed, *ICH* intracerebral haemorrhage, *ICP* intracranial pressure, *IPC* intermittent pneumatic compression, *iv* intravenously, *IVH* intraventricular haemorrhage, *MAP* mean arterial pressure, *MRI/MRA* magnetic resonance imaging/magnetic resonance angiography, *NeuroICU* neurointensive care unit, *NIHSS* National Institutes of Health Stroke Scale/Score, *PaCO*_*2*_ arterial partial pressure of carbon dioxide, *SaO*_*2*_ arterial oxygen saturation, *SBP* systolic blood pressure, *SIADH* syndrome of inappropriate secretion of antidiuretic hormone, *SPECT* single-photon emission computed tomography, *T* temperature, *VTE* venous thromboembolism, *WFNS* World Federation of Neurosurgical Societies

Figure 3 summarises a possible approach for the management of SAH patients in poor neurological condition. The key management of patients with acute brain injury, including the SAH population, is the minimisation of a complex cascade of ischaemic and apoptotic cellular events, oedema, and excitotoxicity that can result in delayed and often progressive secondary brain injury. Unlike primary injury, this delayed damage is considered, at least partially, preventable or reversible if adequately treated. Its prevention, timely detection, and appropriate management require an early, aggressive, and well-structured approach to patient care. This is especially true in patients with poor-grade SAH, where limited neurological examination and a higher incidence of systemic complications make DCI identification a significant challenge.

DCI is often a diagnosis of exclusion; confounding factors such as hypoxia, electrolyte disturbances, infection, fever, hydrocephalus, convulsive, and non-convulsive seizures can produce delayed neurological deterioration similar to that of DCI and should always be considered in the differential and treated accordingly. Moreover, in the poor-grade SAH population, new neurological deficits are clinically difficult to detect because of decreased level of consciousness and the frequent need for sedation (usually required for ICP and mechanical ventilation management), making the detection of acute neurological deterioration even more challenging. Patients who require sedation but who are clinically stable (i.e., absence of ICP crisis, cardiopulmonary instability, or status epilepticus) should undergo interruption of sedation and analgesia (i.e., neurological wake-up tests) that could detect focal neurological deficits. Wake-up tests seem to be safe since they are not associated with changes in cerebral metabolism or oxygenation as measured by microdialysis and direct brain tissue oxygenation measurement, respectively [86]. However, the sensitivity of neurological examination to detect signs of DCI in the setting of poor-grade SAH is low [87]; approximately 20 % of patients who develop DCI, as identified by new infarctions on CT or magnetic resonance, do not have any evidence of clinical neurological deterioration [88, 89]. Interestingly, these patients who developed “asymptomatic” cerebral infarctions were less likely to receive vasopressor agents and had higher frequency of death or moderate-to-severe disability than those with “symptomatic” DCI [88].

**Fig. 4:**
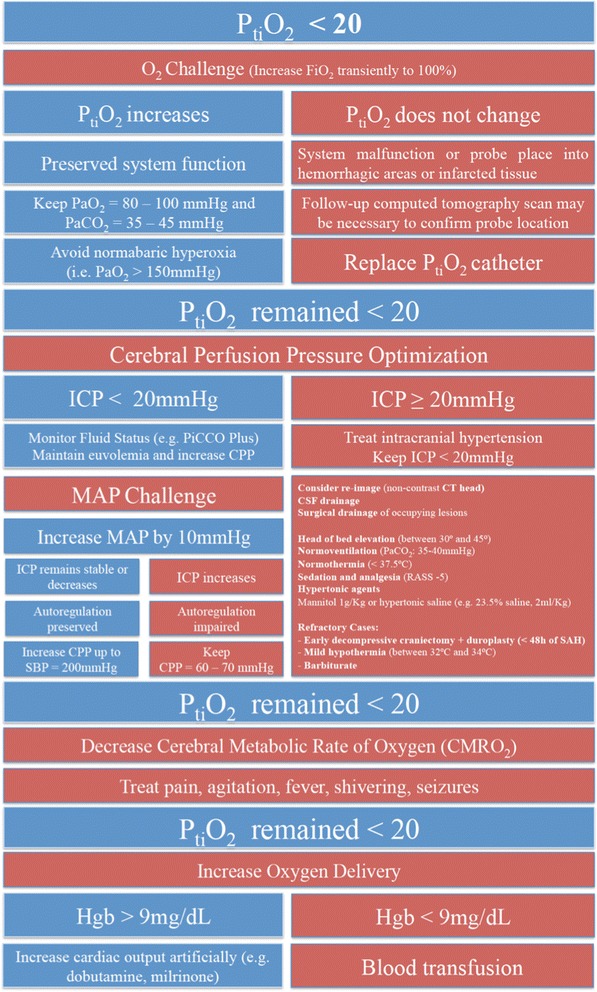
Approach to low brain tissue oxygen. Consider the combined used of P_ti_O_2_ and microdialysis catheter to detect non-hypoxic patterns of cellular dysfunction [97]. According to the manufacturer, an equilibrium time as long as 2 hours may be necessary before P_ti_O_2_ readings are stable, because of the presence of the tip surrounding microhaemorrhages. Sensor damage may also occur during insertion. Increase inspired fraction of oxygen (FiO_2_) to 100 %. If P_ti_O_2_ increases, it confirms good catheter function. Oxygen challenge to assess tissue oxygen reactivity. FiO_2_ is increased from baseline to 100 % for 5 minutes to evaluate the function and responsiveness of the brain tissue oxygen probe. A positive response happens when P_ti_O_2_ levels increase in response to higher FiO_2_. A negative response (lack of P_ti_O_2_ response to higher FiO_2_) suggests probe or system malfunction. Another possibility if there is a negative response is that the probe placement is in a contused or infarcted area. Follow-up computed tomography might be necessary in this situation to ensure appropriate probe position. Mean arterial pressure (MAP) challenge to assess cerebral autoregulation. MAP is increased by 10 mm Hg. Patients with impaired autoregulation demonstrated an elevation in ICP with increased MAP. When the autoregulation is intact, no change or a drop in ICP levels follows the elevation in blood pressure. Another way to assess cerebral autoregulation is the evaluation of the index of P_ti_O_2_ pressure reactivity. When autoregulation is intact, P_ti_O_2_ is relatively unaffected by changes in CPP, so the index of P_ti_O_2_ pressure reactivity is near zero [170]. The threshold haemoglobin (Hgb) of 9 mg/dl to indicate blood transfusion was based on a previously published P_ti_O_2_ study [171]. *CPP* cerebral perfusion pressure, *CSF* cerebrospinal fluid, *CT* computed tomography, *ICP* intracranial pressure, *PaCO*_*2*_ arterial partial pressure of carbon dioxide, *PaO*_*2*_ partial pressure of oxygen in arterial blood, *P*_*ti*_ *O*_*2*_ brain tissue oxygen pressure, *RASS* Richmond Agitation-Sedation Scale, *SAH* subarachnoid haemorrhage, *SBP* systolic blood pressure

Because neurological examination is less useful in this setting, a suspicion of DCI will frequently be based on changes detected by screening tools. According to the Neurocritical Care Guidelines on the management of SAH, “in sedated or poor-grade SAH patients, clinical deterioration may be difficult to assess, and transcranial Doppler (TCD), continuous electroencephalography (cEEG), brain tissue oxygen pressure (P_ti_O2) monitoring, and/or cerebral microdialysis (CMD) are options for monitoring for vasospasm and DCI”. Changes commonly used to trigger intervention include the following [8, 90]:

An increase in either (a) TCD mean flow velocity in the middle cerebral artery (FVMCA) of more than 50 cm/second over 24 hours or (b) mean FVMCA of at least 200 cm/second or middle cerebral artery/internal carotid artery ratio of more than 6 or both [8].
2. CT perfusion parameters: CBF of less than 25 ml/100 g/minute or mean transit times (MTTs) of more than 6.5 seconds or both [91].
Severe angiographic vasospasm (defined as a narrowing of at least 70 % from baseline) [92] detected by digital subtraction angiography (i.e., gold standard) or CT angiography (which is also highly specific for angiographic vasospasm).
Electroencephalography (EEG) reduced alpha variability [93].
Abnormal levels of brain tissue oxygen (P_ti_O_2_ of less than 20 mm Hg; Fig. 4) or CMD (i.e., lactate/pyruvate ratio (LPR) of more than 40 and glucose of less than 0.5 mM and in second line for glutamate of more than 40 mM) or both [93].

#### Multimodal neuromonitoring

Modalities capable of monitoring CBF (e.g., CT perfusion or CTP), cerebral oxygenation (e.g., brain tissue oxygen catheter), and cerebral metabolism (e.g., microdialysis) are theoretically superior to modalities monitoring exclusively vessel diameter (e.g., TCD, conventional angiography, and CT angiography, or CTA). We have previously published a possible approach combining the use of TCD and multimodal CT [94, 95] for monitoring patients with SAH in accordance with the VASOGRADE [6]. It is important to mention that, in the poor-grade population, if screening CTA or digital subtraction angiography has already recognised the presence of severe angiographic vasospasm in a setting of acute neurological deterioration, it is reasonable to start empiric DCI therapy without additional neurological investigation. Additionally, when screening CTP demonstrates perfusion deficits (CBF of less than 25 ml/100 g/minute or MTT of more than 6.5 seconds or both) [91], it is reasonable to initiate therapy for DCI.

ICP and cerebral perfusion pressure (CPP) monitoring have been the cornerstone parameters in the management of comatose patients with acute brain injury. Critical levels of CPP (of less than 70 mm Hg) have been significantly associated with cerebral infarction [96] after SAH. Also, CPP of less than 60 mm Hg has been associated with higher ICP levels and abnormal levels of P_ti_O_2_ and LPR [97]. However, recent clinical data suggest that cerebral hypoxia (P_ti_O_2_ of less than 20 mm Hg) and cerebral energy dysfunction (LPR of more than 40) may occur despite normal levels of ICP and CPP in the poor-grade SAH population [97].

Chen et al. [97], in a cohort of 19 patients with poor-grade SAH, demonstrated that ICP and CPP monitoring may not be sufficient to detect episodes of cerebral compromise, such as severe brain hypoxia detected by P_ti_O_2_ catheter (P_ti_O_2_ of not more than 10 mm Hg) or brain energy dysfunction detected by CMD (LPR of at least 40). The sensitivities of abnormal ICP or CPP levels for elevated LPR and reduced P_ti_O_2_ were 21.2 %, and critical levels of LPR or P_ti_O_2_ were found on many occasions when ICP or CPP was normal. Additionally, early brain tissue hypoxia (i.e., within 24 hours of haemorrhage) is very prevalent in the poor-grade SAH population [98]. Therefore, the use of multimodal neuromonitoring may be a good complement to ICP/CPP monitoring, which could detect cerebral oxygen or energy compromise in an early reversible state [93] (Fig. 4).

#### Continuous electroencephalography monitoring in patients with poor-grade subarachnoid haemorrhage

Continuous EEG (cEEG) has been described as a useful monitoring tool for the prediction and diagnosis of angiographic vasospasm and DCI. Also, cEEG findings may be a prognostic marker in patients with poor-grade SAH [99, 100]. Several studies have investigated and demonstrated a positive correlation between cEEG findings and angiographic vasospasm, DCI, and functional outcome [99–102], supporting the critical care use of this modality in poor-grade or sedated SAH patients. Commonly described quantitative cEEG findings that predict angiographic vasospasm or DCI are (a) decreased relative alpha variability [101] and (b) decreased alpha/delta ratio [100, 102]. Other cEEG findings such as periodic epileptiform discharges, electrographic status epilepticus, and the absence of sleep architecture have been described as independent prognostic factors in the poor-grade SAH population after adjustment for known prognostic factors such as age, clinical grade (i.e., Hunt and Hess grade), and the presence of intraventricular haemorrhage [99]. Claassen et al. [99] described, in a cohort of 116 patients with SAH, that the absence of sleep architecture (80 % versus 47 %; OR 4.3, 95 % CI 1.1–17.2) and the presence of periodic lateralised epileptiform discharges (PLEDs) (91 versus 66 %; OR 18.8, 95 % CI 1.6–214.6) were associated with 3-month poor outcome by modified Rankin scale. Additionally, all patients with absent EEG reactivity, generalised periodic epileptiform discharges, and bilateral independent PLEDs and 92 % of patients (11 out of 12) with non-convulsive status epilepticus progressed to have a poor functional outcome at 3 months.

#### Monitoring brain tissue partial pressure of oxygen

The invasive monitoring of brain tissue oxygenation allows regional and continuous monitoring of P_ti_O_2_, which may detect early changes in cerebral tissue oxygenation that precede ischaemic damage. P_ti_O_2_ levels of below 20 mm Hg require attention and might be a warning sign of ischaemia not detected clinically. P_ti_O_2_ levels of below 15 mm Hg require immediate intervention to optimise cerebral tissue oxygenation (Fig. 4). P_ti_O_2_ levels have been directly correlated with the development of ischaemic events [96], angiographic vasospasm [103], and outcome [104]. In addition to P_ti_O_2_ monitoring, the use of CMD may be a possible alternative for monitoring sedated or poor-grade patients at risk of DCI. The combined use of P_ti_O_2_ and CMD catheter can help discriminate two patterns of cellular dysfunction (i.e., hypoxic and non-hypoxic cellular dysfunction) [97].

CMD measures the interstitial levels of several substances, such as glucose, lactate, pyruvate, glutamate, glycerol, and several inflammatory biomarkers. An increased LPR is the most common and better-studied marker of anaerobic cerebral metabolism and therefore is an indicator of cerebral ischaemia [93]. Metabolic changes detected by CMD, such as elevated LPR, have been shown to predict delayed neurological deterioration and “symptomatic vasospasm” [105, 106]. Also, extreme microdialysate values of lactate, glutamate, LPR, and glycerol have been associated with cerebral infarction and permanent neurological deficits [107].

### Pharmacological prophylaxis

Table 3 summarises drugs investigated and under investigation for prevention of DCI. According to the American Heart Association, the Neurocritical Care Society, and the European guidelines [8–10], nimodipine, an L-type dihydropyridine calcium channel antagonist, is the only medication proven to improve outcomes after SAH [108]. The concept that nimodipine decreases the rate of angiographic vasospasm has been challenged, and the mechanisms by which it improves patient outcome in a setting of SAH are not completely established.

**Table 3:** Evidence review of drugs used in aneurysmal subarachnoid haemorrhage

Drug	Direct drug action	Possible mechanisms of action	Status	Guidelines [8–10]
Nimodipine [82]	L-type calcium channel antagonist	• Reduction of angiographic vasospasm • Increase in fibrinolytic activity • Neuroprotection • Inhibition of cortical spreading ischaemia	Meta-analysis of clinical trials found that oral nimodipine reduced the risk of DCI and poor outcome.	Class I, level A Nimodipine should be administered enterally (60 mg every 4 hours) to prevent DCI. The only drug approved for SAH in the USA and Europe.
Clazosentan [168]	Endothelin A receptor antagonist	Reduction of angiographic vasospasm	• Four randomised clinical trials and a meta-analysis • Clazosentan reduced angiographic vasospasm without a significant effect on outcome. • Hypotension and pulmonary complications associated with the drug use could have counteracted the beneficial effects of the drug.	Not addressed However, after the publication of the CONSCIOUS trials and following meta-analysis, clazosentan infusion will not be recommended for patients with SAH, as a Class I, level A.
Fasudil [172]	Rho-kinase inhibitor	Reduces smooth muscle contraction and inhibits TNF-induced IL-6 release from C6 glioma cells	• Eight randomised clinical trials • Treatment significantly reduced the incidence of angiographic vasospasm and cerebral infarction and improved the odds ratio for good recovery compared with placebo or nimodipine and other drugs.	Not addressed The drug is approved for use in patients in Japan and China but not in Europe or USA.
Statins [92–94]	Inhibit HMG-CoA reductase	• Preserve endothelial function • Anti-inflammatory effects • Antioxidant • Antithrombotic actions • Vascular protection • Neuroprotective and neurorestorative action	• Seven randomised clinical trials of statins in patients with SAH. • An additional study showing no benefit of higher dose of simvastatin (80 mg versus 40 mg) • One systematic review not including the STASH trial found no effect of statin treatment on poor outcome.	Guidelines published before the STASH trial [92]. The recommendations will probably remain the same to administer statins only if the patient was already receiving them at time of SAH, as a Class I, level A.
Magnesium [90]	Antagonism of calcium channels on vascular smooth muscle	• Vasodilation • Increased endothelial cell prostacyclin • Endothelial protection • Protect the blood–brain barrier • Reduce cerebral oedema • Anticonvulsant (*N*-methyl-d-aspartate receptor antagonism)	• Seven randomised clinical trials • Meta-analysis reported no effect of magnesium on poor outcome	Class I, level A Magnesium is not recommended for prevention of DCI.
Dantrolene [173]	Inhibits ryanodine receptors	Reduces intracellular calcium release in smooth muscle and may be neuroprotective	• One small dose-escalation study • Dantrolene in a dose of 2.5 mg/kg, administered over the course of 60 minutes, was associated with reduced cerebral blood-flow velocities measured by transcranial Doppler.	Not addressed Remains experimental
Intrathecal thrombolytics (i.e., urokinase and recombinant tissue plasminogen activator) [174]	Fibrinolytic agents	The rapid clearance of subarachnoid clot could reduce angiographic vasospasm and complications, such as cortical spreading ischaemia and microthrombosis.	• Five RCTs and a meta-analysis • Thrombolysis was associated with significant reductions in angiographic vasospasm, delayed neurological deficits, hydrocephalus, and poor outcome.	Not addressed Further trials are needed. Standardisation of techniques and evaluation in a larger study are required.
Antiplatelet drugs [175] • Acetylsalicylic acid • OKY-046 (Cataclot) - selective thromboxane synthetase inhibitor • Dipyridamole • Ticlopidine	Inhibition of platelet aggregation	Inhibition of platelet aggregation	• Seven randomised clinical trials and a meta-analysis found trends toward reduction in poor outcome but also toward increased intracranial haemorrhage. • Only ticlopidine was associated with statistically significant fewer occurrences of a poor outcome (only one small RCT)	Not addressed Further trials are needed. According to the meta-analysis results, treatment with antiplatelet agents to prevent DCI or poor outcome cannot be recommended.
Albumin [176]	Multiple	Neuroprotective	• One open-label dose-escalation trial • Trend toward improved outcome with 1.25 g/kg per day	Not addressed Remains experimental
Erythropoietin [177, 178]	Multiple	• Prevent loss of autoregulation • Reduce angiographic vasospasm • Inhibits apoptosis and stimulates neurogenesis and angiogenesis	• Two RCTs • One negative study and one showing that patients who received erythropoietin had fewer cerebral infarcts, shorter duration of autoregulatory dysfunction, and better clinical outcome.	Not addressed Remains experimental
Cilostazol [179]	Inhibits phosphodiesterase 3	• Antithrombotic • Vasodilatory • Anti-smooth muscle proliferation • Inotropic and chronotropic effects	• One small (109 patients) randomised, single-blind study • Cilostazol significantly reduced angiographic vasospasm, DCI, and cerebral infarction but had no effect on outcome.	Not addressed Remains experimental

*CONSCIOUS* Clazosentan to Overcome Neurological Ischaemia and Infarction Occurring After Subarachnoid Haemorrhage, *DCI* delayed cerebral ischaemia, *IL-6* interleukin-6, *RCT* randomised controlled trial, *SAH* subarachnoid haemorrhage, *STASH* simvastatin in aneurysmal subarachnoid haemorrhage, *TNF* tumour necrosis factor

Nimodipine probably has a neuroprotective action by decreasing the influx of calcium after cerebral ischaemia due to DCI. Additionally, nimodipine might decrease the incidence of microthrombi by increasing the endogenous fibrinolysis [109] and may antagonise cortical spreading ischaemia [110]. Nimodipine seems to improve long-term outcome in the poor-grade population as well [111]. A multicentre, randomised placebo-controlled double-blind trial studied the effect of nimodipine in 188 patients with poor-grade SAH (Hunt and Hess grade 3–5) [111]. The treatment was associated with an improvement in functional outcome at 3 months (29.2 % in the nimodipine group versus 9.8 % in the placebo), despite similar rates of moderate and severe angiographic vasospasm found in the follow-up angiography (64.3 % in the nimodipine group versus 66.2 % in the placebo group). However, in the sub-group of grade 5 patients, no difference in functional outcome between nimodipine and placebo groups was found [111].

Interestingly, in the poor-grade population, the administration of nimodipine is associated with an acute drop in the mean arterial pressure and CPP, which is translated into a decrease in CBF and brain tissue oxygenation [112, 113]. However, there is no prospective study that evaluates the long-term consequences of these physiological changes on functional outcome.

#### Magnesium

Magnesium is a calcium channel antagonist with potent vasodilator and neuroprotective properties. Animal models of SAH have shown reversal of cerebral arterial vasoconstriction, leading to reduction of the size of ischaemic lesions [114]. Additionally, magnesium may decrease the rate and frequency of cortical spreading ischaemia [115]. Unfortunately, a large clinical trial combined with a meta-analysis [116] showed no clinical benefit with the use of magnesium infusion, measured as favourable outcome at 6 months, incidence of DCI, or cerebral infarction. A possible explanation is that high levels of plasma magnesium are associated with worse clinical outcomes [117].

#### Statins

There is great interest in the impact of statins in the prevention of DCI. Statins preserve endothelial function by increasing nitric oxide synthesis while decreasing the synthesis of endothelin-1. Also, there are other statin effects that may be interesting in the SAH setting, such as anti-inflammatory, antioxidant, and antithrombotic effects. Additionally, statins have described neuroprotective and neurorestorative action. So far, six randomised clinical trials [118] of statins in patients with SAH have been published; however, a systematic review of these studies found no effect of statin treatment on poor outcome; mortality was 10 % in the statin group versus 21 % in controls (relative risk 0.62, 95 % CI 0.36–1.06); DCI was significantly reduced in the statin group. The overall quality of these studies was judged to be low to moderate. Recently, two multicentre randomised clinical trials were published. One compared two different regimens of simvastatin (80 versus 40 mg), which showed no effect of higher dose on DCI, modified Rankin disability score at 3 months, and an analysis of cost-effectiveness [119]. The second study had previously shown no benefit in the use of 40 mg simvastatin compared with placebo for long-term outcome, as measured by modified Rankin score at 6 months [120]. Mortality and favourable outcome were similar in both simvastatin and placebo groups (10 % versus 9 % and 58 % versus 62 %, respectively). Serious adverse events were also similar in both groups (18 %) [120]. Therefore, the guidelines will probably keep their recommendation to administer statins only if the patient was already receiving them at the time of SAH [118].

### Haemodynamic prophylaxis

The use of prophylactic hypervolemia, a component of so-called triple-H therapy (hypervolemia, hypertension, and haemodilution), is not recommended [8–10], based on lack of evidence that it positively affects functional outcome. It also increases the costs and risk of systemic complications, such as cardiac dysfunction, pulmonary oedema, and infection [121, 122].

#### Delayed cerebral ischaemia treatment

Haemodynamic manipulation, what is known as the triple-H therapy, has for decades been the cornerstone of DCI management [94, 95]. However, the literature supporting its safety and efficacy is scarce [123]. Angiographic vasospasm, in the absence of DCI, should not be treated [90, 124]. The development of a new focal deficit or a decrease in level of consciousness, not explained by other causes (e.g., hydrocephalus or re-bleeding), should prompt aggressive treatment [90, 124]. A fluid bolus with normal saline might be the first step because it increases CBF in areas of cerebral ischaemia [125]. The main goal is to maintain euvolemia and normal circulating blood volume. Hypervolemia and haemodilution do not improve cerebral oxygen delivery and may be associated with adverse events [121, 122]. Patients who fail to completely reverse the new deficit after a fluid challenge may undergo a trial of hypertension unless the blood pressure is elevated at baseline or in the presence of heart failure [9]. Blood pressure is augmented in a step-wise fashion by the use of a vasopressor, typically noradrenaline [8, 126]. The neurologic examination is repeated frequently in each blood pressure step (180 mm Hg/190 mm Hg/200 mm Hg), and the target should be based on clinical improvement. If the neurological deficit persists after the induction of hypertension (typically up to a systolic blood pressure of 200 to 220 mm Hg), a rescue therapy with cerebral angioplasty or intra-arterial infusion of a vasodilator might be of benefit [127]. The prophylactic use of angioplasty is not associated with improved outcome and might be associated with increased risk of arterial rupture and is not recommended [128].

## Medical complications

It is well described that medical complications after SAH have a negative impact on survival and functional outcome. Up to 80 % of patients will develop a serious medical complication during phase 2, increasing the risk for secondary brain injury [129].

Cardiac complications following SAH can range from benign electrocardiogram changes to overt cardiogenic shock requiring intra-aortic balloon pump [130, 131]. Positive troponin is a good marker of left ventricular dysfunction after SAH [132], which increases the risk of hypotension, pulmonary oedema, and cerebral infarction [133]. The treatment is mainly supportive, and most of the cases will recover spontaneously within 2 weeks [134]. However, aggressive ICU management may be required in the setting of severely impaired left ventricular function and DCI. Thus, the use of inotropic agents such as dobutamine [135], levosimendan [136], milrinone [137], and even intra-aortic balloon pump counterpulsation [138] has been described and can be considered to optimise the cardiac function in order to improve CBF.

Patients with poor-grade SAH are at higher risk of cardiac and pulmonary complications [139]. Additionally, hypovolemia and pulmonary oedema are common phenomena in this population, increasing the risk for delayed cerebral ischaemia [140, 141]. Therefore, the poor-grade SAH population may benefit from advanced haemodynamic monitoring. Yoneda et al. [139], in a multicentre prospective cohort study of haemodynamic monitoring using a transpulmonary thermodilution system (PiCCO Plus), which included a group of 138 patients with poor-grade SAH, showed that extravascular lung water index (*P* = 0.049), pulmonary vascular permeability index (*P* = 0.039), and systemic vascular resistance index (*P* = 0.038) were significantly higher in the poor-grade group when compared with the good-grade population. Additionally, poor-grade patients displayed significantly lower cardiac index on days 1 and 2 (*P* = 0.027 and *P* = 0.011, respectively) and developed heart failure-like afterload mismatch at an early stage, and those who developed DCI had haemodynamic measures of hypovolemia, as shown by a decreased global end-diastolic volume index [139]. The same group described the mean global end-diastolic volume index (normal range, 680–800 ml/m^2^) as an independent factor for the development of DCI (HR 0.74, 95 % CI 0.60–0.93; *P* = 0.008). Patients who developed DCI had significantly lower global end-diastolic volume index compared with patients who did not (783 ± 25 ml/m^2^ versus 870 ± 14 ml/m^2^; *P* = 0.007). A threshold of less than 822 ml/m^2^ was correlated with DCI development, whereas a global end-diastolic volume index above 921 ml/m^2^ was associated with the development of severe pulmonary oedema. These finding suggest that maintaining global end-diastolic volume index slightly above the normal range may be effective to prevent hypovolemia and severe pulmonary oedema, which may decrease the risk of DCI.

Pulmonary complication, such as hospital-acquired pneumonia, cardiogenic or neurogenic pulmonary oedema, aspiration pneumonitis, and pulmonary embolism, occur in approximately 30 % of patients after SAH [142]. Acute respiratory distress syndrome can affect 27 % of cases and is independently associated with worse outcomes [143]. In this clinical scenario, extra caution should be taken to avoid fluid overload; however, diuretics might be dangerous because of the risk of hypovolemia-induced cerebral ischaemia.

Hyponatremia (serum sodium of less than 135 mEq/dl) is the most common electrolyte derangement after SAH, occurring in up to 50 % of patients. There are two possible mechanisms responsible for the development of hyponatremia after SAH: (1) cerebral salt wasting (CSW) and (2) the syndrome of inappropriate secretion of antidiuretic hormone (SIADH) [144]. These entities are fundamentally different in their pathogenesis; however, they are difficult to distinguish in clinical practice and may concur in the same patient [145]. Importantly, CSW courses with intravascular volume contraction, which increases the risk of DCI and poor outcome [145]. Likewise, the treatment of SIADH on the basis of fluid restriction is not indicated, because of increased risk of hypovolemia-associated cerebral infarction [146, 147]. Therefore, in clinical practice, the management of hyponatremia in the setting of SAH is based on the avoidance of hypovolemia and the judicious repletion of volume and sodium losses [144].

In a retrospective study in a single academic centre, Wartenberg et al. found that a single occurrence of hyperglycaemia, fever, or anaemia after aneurismal SAH was independently predictive of poor outcome, even after adjustment for traditional prognostic variables, such as age, clinical grade, aneurysm size, re-bleeding, and cerebral infarction [129].

Fever is the most common medical complication after SAH and is associated with longer ICU and hospital length of stays, worse functional outcomes, and higher mortality [148, 149]. Although non-infectious fever is common, especially in the presence of intraventricular haemorrhage and poor-grade patients [150], it is strongly recommended that frequent temperature checks and careful assessment for possible infectious cause are made. During the time window of vasospasm, it is desirable to maintain normothermia with antipyretic drugs, followed by advanced fever control with surface cooling or intravascular devices [151, 152]. In this situation, especial attention should be paid to detect and treat shivering. The protocol for diagnosis and treatment of shivering has been published elsewhere [153].

Ideally, blood sugar should be kept less than 200 mg/dl and hypoglycaemia (less than 80 mg/dl) should be strictly avoided. Both have been shown in microdialysis studies to be associated with metabolic crisis and worse neurological outcome [154, 155].

Anaemia can be easily corrected, but blood transfusion has been implicated with worse outcome after SAH [156, 157], including higher mortality, after adjustment for the most common clinical indications of transfusion [158].

Although there is no clear threshold for transfusion in patients with SAH, general ICU thresholds are not applicable for this population [7, 101, 159]. Dhar et al. [160], in an elegant study using positron emission tomography scan, demonstrated that transfusion in patients with haemoglobin levels of less than 9 g/dl was the only intervention capable of increasing global CBF and oxygen delivery, when compared with crystalloid bolus and induced hypertension. The clinical applicability of these findings needs to be addressed in a large trial because the study enrolled a small number of patients (38 in total) and had only physiological endpoints.

Patients with poor-grade SAH are at high risk of venous thromboembolism [161]. Guidelines on management of SAH suggest starting mechanical prophylaxis with intermittent compression devices before aneurysm treatment [8–10]. Pharmacologic thromboprophylaxis seems to be safe if started within 12 to 24 hours after aneurysm treatment [162].

## Conclusions

Aneurysmal SAH is a complex neurovascular disease associated with multiple neurological and systemic complications and requires multidisciplinary specialised care, best provided in high-volume centres. Patients who survive the initial bleed can deteriorate within 2 weeks, especially because of DCI. DCI is a syndrome with a complex multifactorial pathophysiology that extends beyond the historic explanation of angiographic vasospasm.

Although the risk of death and moderate and severe disability has decreased significantly over the last three decades, the extent to which the prevalence of cognitive deficits, poor quality of life, and day­to­day functioning have changed over the same period is completely undetermined. Further studies should simultaneously tackle multiple pathophysiological pathways, and long-term functional outcomes shall be assessed by means of outcome measures able to recognise subtle cognitive changes.

## Abbreviations

CBF: Cerebral blood flow
cEEG: Continuous electroencephalography
CI: Confidence interval
CMD: Cerebral microdialysis
CPP: Cerebral perfusion pressure
CSF: Cerebrospinal fluid
CSI: Cortical spreading ischaemia
CSW: Cerebral salt wasting
CT: Computed tomography
CTA: Computed tomography angiography
CTP: Computed tomography perfusion
DCI: Delayed cerebral ischaemia
EBI: Early brain injury
EEG: Electroencephalography
EVD: External ventricular drain
FVMCA: Flow velocity in the middle cerebral artery
GCS: Glasgow Coma Scale
HR: Hazard ratio
ICP: Intracranial pressure
ICU: Intensive care unit
LPR: Lactate/pyruvate ratio
MTT: Mean transit time
OR: Odds ratio
PaCO_2_: Arterial partial pressure of carbon dioxide
PLED: Periodic lateralised epileptiform discharge
P_ti_O_2_: Brain tissue oxygen pressure
SAH: Subarachnoid haemorrhage
SIADH: Syndrome of inappropriate secretion of antidiuretic hormone
TBI: Traumatic brain injury
TCD: Transcranial Doppler
WFNS: World Federation of Neurosurgical Societies.
